# Short-Range Responses of the Kissing Bug *Triatoma rubida* (Hemiptera: Reduviidae) to Carbon Dioxide, Moisture, and Artificial Light

**DOI:** 10.3390/insects8030090

**Published:** 2017-08-29

**Authors:** Andres Indacochea, Charlotte C. Gard, Immo A. Hansen, Jane Pierce, Alvaro Romero

**Affiliations:** 1 Department of Entomology, Plant Pathology and Weed Science, New Mexico State University, Las Cruces, NM 88003, USA; aindacoc@gmail.com; 2 Department of Economics, Applied Statistics, and International Business, New Mexico State University, Las Cruces, NM 88003, USA; cgard@nmsu.edu; 3 Department of Biology, New Mexico State University, Las Cruces, NM 88003, USA; immoh@nmsu.edu; 4 Department of Entomology, Plant Pathology and Weed Science, New Mexico State University, Artesia, NM 88210, USA; japierce@ad.nmsu.edu

**Keywords:** kissing bugs, insect behavior, host seeking, EthoVision, olfactometer, relative humidity, light attraction, carbon dioxide

## Abstract

The hematophagous bug *Triatoma rubida* is a species of kissing bug that has been marked as a potential vector for the transmission of Chagas disease in the Southern United States and Northern Mexico. However, information on the distribution of *T. rubida* in these areas is limited. Vector monitoring is crucial to assess disease risk, so effective trapping systems are required. Kissing bugs utilize extrinsic cues to guide host-seeking, aggregation, and dispersal behaviors. These cues have been recognized as high-value targets for exploitation by trapping systems. A modern video-tracking system was used with a four-port olfactometer system to quantitatively assess the behavioral response of *T. rubida* to cues of known significance. Also, response of *T. rubida* adults to seven wavelengths of light-emitting diodes (LED) in paired-choice pitfall was evaluated. Behavioral data gathered from these experiments indicate that *T. rubida* nymphs orient preferentially to airstreams at either 1600 or 3200 ppm carbon dioxide and prefer relative humidity levels of about 30%, while adults are most attracted to 470 nm light. These data may serve to help design an effective trapping system for *T. rubida* monitoring. Investigations described here also demonstrate the experimental power of combining an olfactometer with a video-tracking system for studying insect behavior.

## 1. Introduction

Kissing bugs (Hemiptera: Reduviidae) are blood-sucking insects of considerable significance to human health, especially in the Americas. In addition to having a bite that can cause serious allergic reactions in some people, kissing bugs are vectors of Chagas disease or American trypanosomiasis [1,2]. The economic and medical impact of this disease is underappreciated and it is one of the most neglected tropical diseases in the world [3]. In kissing bugs, the prevalence of the causative agent, *Trypanosoma cruzi* Chagas, is maintained by sylvatic and peridomestic cycles involving a variety rodents and other endothermic vertebrates, including pets [4]. Eradication of the disease is a dubious proposition and places more importance on vector monitoring and control. Additionally, kissing bugs threaten to spread Chagas disease to non-endemic areas where they are present. *Triatoma rubida* Laporte is considered an important potential vector of Chagas in the southwestern United States and Northern Mexico [5,6].

Public health problems presented by kissing bugs in the United States are being exacerbated by the rapid expansion of human populations into kissing bug habitats [7,8]. In southern Arizona, kissing bugs live primarily in close association with the white-throated woodrat, *Neotoma albigula* [9]. From late May through mid-July, hungry kissing bugs leave the nests of the white-throated woodrats and begin dispersal flights [7,10,11]. During this flight, bugs may invade human domiciles, a process mediated by their attraction to artificial light. Homes with an abundance of light sources (porch and patio lights or light shining through windows) are more likely to be invaded [7,10,11,12]. At least seven species of kissing bugs have been found inside houses in 13 southern states [13]. In a survey of a suburban area in Southern California, 7% of responders reported triatomine sightings in their house while another 7% indicated that they were bitten by a triatomine [14]. Bites can produce in humans mild to strong local and general anaphylactic reactions [1,15]. Attempts to reduce the number of kissing bugs are made by spraying residual insecticides in and around homes [16] and by making structural modifications to reduce colonizations and intrusions of insects into homes [17]. *Triatoma rubida* is frequently found in Arizona, New Mexico [7,10,11,13], and southwest Texas [18]. Analysis on the blood meals of *T. rubida* collected in Arizona indicates that this species feeds regularly on humans [19]. This species has been shown to be infected with *T. cruzi* at a significant rate in New Mexico (66%) [20]. *Triatoma rubida* are found to have a similar prevalence of *T. cruzi* in the neighboring states of Texas (61.5%) [18] and Arizona (41.5%) [10]. Female *T. rubida* seem to be particularly good vectors of the protozoan, as they consistently defecate while feeding [6,21]. The attraction of *T. rubida* to lights and their subsequent invasion of human environments has important epidemiological significance because it enhances the possibility of contact between these insects and humans. Detailed information on insect distributions in the Southern United States is crucial to disease risk assessment efforts.

In order to properly study the distribution of an insect, an effective trapping system is needed. Traps that have been developed to attract insects commonly exploit some aspect of that insect’s behavior [22,23]. Like other hematophagous insects, kissing bugs respond to an array of environmental cues to locate and identify potential hosts. Odorants like carbon dioxide (CO_2_), isobutyric acid, and l-lactic acid are host-associated cues that have been shown to have synergistic effects when presented simultaneously [24]. At short distances, other host-associated cues like heat and water vapor content play a larger role [25]. Heat is also critical for the initiation of biting and feeding [26]. These kinds of cues have been used with relative success in traps for blood-sucking insects. Carbon dioxide, which is associated with the breath of an endothermic vertebrate, is often employed to attract mosquitoes to funnel-style traps [27] or bed bugs to dog bowl-style traps [23]. Similar traps that use sugar solutions with baker’s yeast as the CO_2_ source have also been tested on kissing bugs [28]. In addition to host-derived cues, light can be an attractive cue to many insects when used in a trap [29]. More elaborate trapping systems have been explored for bed bugs that use a combination of CO_2_, heat, and chemical lures [30]. With better understanding, the response of *T. rubida* to these cues could be exploited in a similar trapping system. In this study, we employed a modern video tracking technique to analyze the responses of *T. rubida* to host-associated cues and detail aspects of this orientation behavior that may be important to public health and monitoring efforts.

## 2. Materials and Methods

### 2.1. Insects

A laboratory colony of *T. rubida* was used for most experiments. The colony was established from adult *T. rubida* specimens collected from a residence in Anthony, New Mexico in the summer of 2013. Kissing bugs were reared in an incubator on a 12:12 light:dark cycle at 25 °C and 60 ± 5% relative humidity. Insects were fed on defibrinated rabbit blood (Hemostat Laboratories, Dixon, CA, USA) in an artificial feeding system similar to that described by Montes et al. [31]. Evaluations of responses to moisture and CO_2_ used fifth-instar nymphs 2–3 weeks post-feeding, likely to seek a host. Light experiments used wild adult males captured in the field 2 to 3 months before being assayed. Like the nymphs, these insects were fed regularly and starved 2–3 weeks prior to testing; they were also kept in the same environmental conditions. The mating status of collected bugs was not known and no method was available to distinguish mated and non-mated bugs. 

### 2.2. Olfactometer and Tracking of Activity

Responses of *T. rubida* to different CO_2_ and humidity levels were tested using an ARS four-port olfactometer (Analytical Research Systems, Gainesville, FL, USA) similar to that used by Milne et al. [24]. The olfactometer is connected with an air delivery system that is supplied by a 200 L cylinder of USP (United States Pharmacopeia) grade oxygen. Airflow was adjusted to approximately 250 mL/min for each port. A fifth flowmeter controlled the flow of the vacuum which was not created by a mechanical pump; instead, it used pressurized gas from the cylinder to create a negative pressure in the vacuum line, which was set to 1 L/min. Different ports were randomly selected as the experimental (treatment) port to minimize bias. Bugs were allowed to acclimate to room conditions for 20 min and then placed directly in the vacuum port that had a piece of manila folder (4.5 × 1 cm) to climb and enter the arena. A trial ended after 20 min or when the insect fell in any of the olfactometer traps. All tests were conducted at room temperature (25 °C, 40 ± 5% relative humidity (RH) during the first six hours of scotophase. Before each trial, the plastic parts of the olfactometer were cleaned with ethanol and rinsed with deionized water; acetone was used to clean the glass.

**Figure 1:**
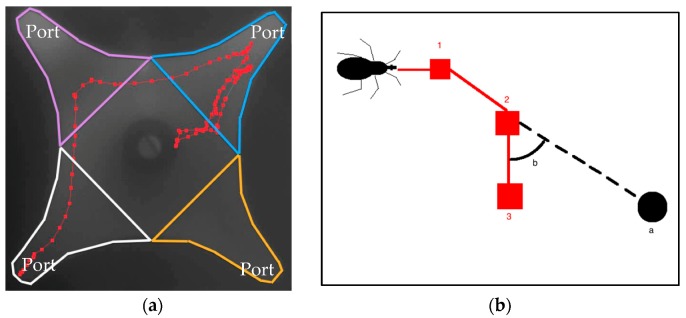
View of arena of the four-port olfactometer. (**a**). Four bell-shaped zones were delimited near olfactometer ports. The activity of individual insects in each zone was captured by a camera and used by EthoVision^®^ XT to generate behavioral parameters; (**b**). The diagram shows how deviation path was calculated in CO_2_ assays. As a kissing bug walks and tracking samples are produced (in red), the mean deviation b from a point of interest a is calculated. The dotted line (in black) represents a zero-deviation path to the point after sample 2.

EthoVision^®^ XT version 11.5 software (Noldus Information Technology Inc., Leesburg, VA, USA) [32] was used to capture and analyze videos of the arena throughout the trial. A NIR (near-infrared) camera (series acA1300-60 gm NIR camera, Basler^®^ ace; Exton, PA, USA), outfitted with a lens (C-mount 4–8 mm varifocal megapixel CCTV lens, model# H2Z0414C-MP, Computar^®^; Torrance, CA, USA) and IR filter (Infrared 850 light filter, Heliopan^®^, North White Plains, NY, USA) was positioned approximately 50 cm directly above the olfactometer arena. Light for the recordings was provided by two IR illuminators (AXTON^®^, North Salt Lake, UT, USA). EthoVision^®^ XT virtually facilitates the division of the arena into four zones in proximity of each port and tracks the movement of bugs in these zones (Figure 1a). Tracking samples from each zone were then used by EthoVision^®^ XT to calculate behavioral variables in each zone, such as the percent of time spent (from the total time insects spent in the whole olfactometer arena), proportion of total time moving (activity), walking velocity, mean deviation, and angular velocity [32]. 

### 2.3. Response to Carbon Dioxide

Carbon dioxide was administered via a gas cylinder with regulatory valves to control flow. Four separate flowmeters were used to regulate the flow of a 200 L tank of 0.5% (5000 ppm) CO_2_ diluted in USP grade oxygen. These four flowmeters mixed air with four flowmeters of the air delivery system and, by adjusting the relative flow of each, the concentrations of CO_2_ of 800, 1600, and 3200 ppm were produced reliably and consistently in the airstreams. A fourth airstream did not carry CO_2_ (0 ppm of CO_2_).

### 2.4. Response to Moisture

Responses of kissing bugs to different levels of moisture was evaluated in the same four-port olfactometer used to test CO_2_. This apparatus has four glass water bubblers or humidifiers placed in-line to humidify an airstream. In order to create airstreams with varying levels of humidity, paper strips (3 by 24 cm) dampened with 100 µL or 1000 µL of deionized water were placed in the bubblers filter to produce 60% and 90% RH airstreams, respectively. A 30% RH airstream was supplied to the olfactometer by delivering air directly from the air tank. A drying column (Drierite desiccant column, W.A. Hammond DRIERITE Co., Xenia, OH, USA) was used to create a relatively dry airstream of 5% RH. An Onset HOBO^®^ UX100 External Temp/RH Data Logger (Onset Computer Corporation, Bourne, MA, USA) was used to measure the relative humidity of the airstream near the port entrances. 

### 2.5. Light Experiments

The methodology was adapted from Diaz-Montano et al. [33] where it was used to test attraction of psocids to light. The arena was two petri dishes on top of each other to form a covered arena (14.5 cm diameter, 3 cm height) with two holes on either side (2 cm in diameter) leveled with the floor of the arena. Holes were large enough to accommodate two short sections of Teflon tubing (1.6 cm inside diameter, 4 cm length) and were used to connect it to two pitfall traps made from amber plastic vials (7.2 cm height, 3.1 cm diameter). Both pitfall traps were covered with black electrical tape to occlude external light. Small holes were drilled in the pitfalls to mount LED (light emitting diode) lights (0.5 cm in diameter) directly opposite of the entrances. When activated, light projected into the arena, and bugs attracted to lights were captured in its respective pitfall. To ensure that bugs were properly acclimated to the arena, they were held in place in the center using a piece of Teflon tubing inserted through a hole in the top of the arena. Lights were connected to a sealed lead-acid battery (SP6-20, 6 V 20AH/NB, SigmasTek, New York, NY, USA) and a potentiometer (RV4NAYSD152A, 2 W Power Rating, 1.5 kO hm Resistance Value, Honeywell International Inc., Morristown, NJ, USA) was used to set the proper voltage for each LED tested. LEDs were chosen to span the visible light spectrum and UV wavelengths: 351 nm (UV, L5-1-U5TH15-1, LEDSupply, Randolph, VT, USA), 390 nm (UV, LED-5U8PK20, Lightobject, Sacramento, CA, USA), 460 nm, 470 nm, 507 nm, 591 nm, 645 nm, and white lights (violet, RL5-V1015; blue, RL5-B2430; green, RL5-G7032; yellow, RL5-Y3230; red, RL5-R1330; white, RL5-W18030, respectively, Super Bright LEDs Inc., St. Louis, MO, USA) were tested. Each choice test consisted of a LED, or white light, and a blank vial as control (no light). Control trials consisted of two vials with no lights. Trials were conducted in dark conditions. Trials were done with groups of five male bugs, and the location of the bugs was recorded 2 h after release. The compositions of groups were randomly selected from a pool of insects, and the placement of active LEDs was randomized in treatment trials. Each trial was repeated between four to six times, and no insect was used twice for the same wavelength, nor in the same day. 

### 2.6. Data Analysis

For evaluations with CO_2_ and moisture in the olfactometer, the number of insects that made a choice were analyzed with a chi-square goodness-of-fit test and compared against an even distribution. The ransacking method was used to compare responses between levels of CO_2_ or moisture [34]. Fifty-one *T. rubida* nymphs were tested in CO_2_ experiments, 40 made choices. These 40 bugs were used for the chi-square analysis. However, all 51 bugs were incorporated for analyses using behavioral parameters from EthoVision^®^.

Lineal data from behavioral parameters were analyzed by means of one-way analysis of variance (ANOVA). Data on the percent of the total time moving (activity) were square root and arcsine transformed before ANOVA and Fisher’s least significant difference (LSD) post-hoc tests (at 5% level of significance). In evaluations with CO_2_, the parameter degrees of deviation of individual insects to CO_2_ airstreams were added by placing reference points at the entrances of the ports and measuring the mean deviation (Figure 1b). This data was analyzed with an ANOVA and Fisher’s LSD post-hoc test. Attraction to light was calculated as an attraction index defined as: AI = (T − C)/(T + C + O), where T = number of males captured in the vial with test light, C = number of males captured in the vials with no light, and O = number of males that remained in the arena [12]. A bootstrap analysis [35] was performed to construct 95% confidence intervals (CIs) for attraction index estimates. Wavelengths were considered significantly more attractive than no light at a 5% significance level if their CIs did not overlap with the CIs for control trials. Collection of data for this experiment was limited by the number of bugs we could collect throughout the season (~150) and the amount of time available to test them (since adults did not live long after mating). All analyses were conducted using SAS software version 9.4 [36].

## 3. Results

### 3.1. Responses to Carbon Dioxide

**Figure 2:**
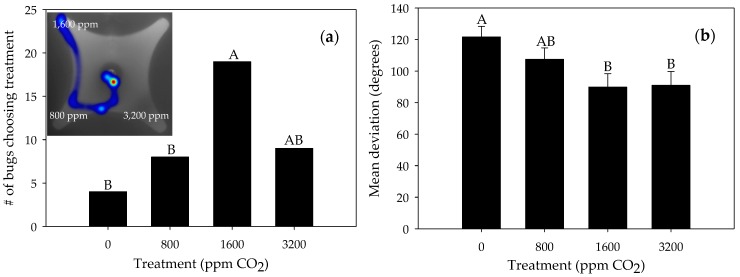
Responses of *Triatoma rubida* nymphs to different concentrations of CO_2_. (**a**) Number of bugs choosing ports with airstreams carrying different levels of CO_2_ (N = 40). EthoVision^®^ generated “heat maps” that visualize a subject’s frequency at specific positions based on a color gradient. The insert represents an example of the trajectory of a *T. rubida* nymph interacting with CO_2_ streams in the olfactometer. In this case, nymphs ventured to other zone before choosing the port with 1600 ppm of CO_2_; (**b**) Mean deviation (degrees) ± Standard Error (SE) of nymphs in each zone near each port (N = 51). Within each panel, bars with the same letter are not significantly different (Ransacking analysis, Fisher’s least significant difference (LSD), *p* > 0.05).

Concentrations of CO_2_ in an airstream significantly affected nymph choices (*χ*^2^ = 12.2, *df* = 3, *p* = 0.0067) (Figure 2a). Nymphs chose 1600 ppm CO_2_ in almost half of all successful trials (19 out of 40 trials), followed by 3200 ppm (9 times), 800 ppm (8 times), and 0 ppm CO_2_ (4 times) (Figure 2a).

Ransacking analyses indicated that 1600 ppm of CO_2_ was chosen significantly more often than 0 and 800 ppm of CO_2_ (*p* = 0.002, *p* = 0.034, respectively), with 3200 ppm CO_2_ not statistically different from either group (Figure 2a). There were no differences in the percent of time spent by insects in each zone (range: 13.4–22%) (F_3,192_ = 1.11, *p* = 0.344), velocity (range 0.29–0.38 cm/s) (F_3,80_ = 0.34, *p* = 0.799), or angular velocity (range: 70.3–86.3 deg/s) (F_3,80_ = 0.40, *p* = 0.755). Carbon dioxide concentrations also significantly affected the degree of orientation of each nymph to the airstreams (F_3,192_ = 3.71, *p* = 0.012) (Figure 2b). The post-hoc analysis indicates bugs deviated significantly less often toward 1600 ppm (89.9 degrees) and 3200 ppm CO_2_ (90.9 degrees) than toward 0 ppm CO_2_ (121.6 degrees). The 800 ppm of CO_2_ treatment with a mean of 107.5 degrees was not statistically different from any other treatment (*p* > 0.05) (Figure 2b). 

### 3.2. Response to Moisture

**Figure 3:**
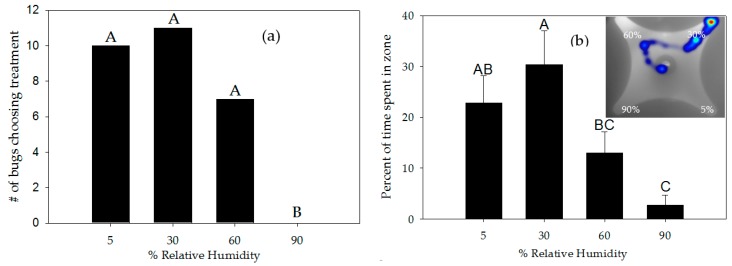
Responses of *Triatoma rubida* nymphs to different levels of relative humidity (RH). (**a**) Bugs choosing ports with airstreams carrying different levels of humidity (N = 28); (**b**) Percent of time (±SE) spent by nymphs in each zone (N = 35 bugs). Photo, the bug sampled 60% then 30% before choosing it. Within each panel, bars with the same letter are not significantly different (Ransacking analysis, Fisher’s LSD, *p* > 0.05).

Airstream moisture significantly affected nymph choices (χ^2^ = 10.5, *df* = 3, *p* = 0.014) (Figure 3a). Ransacking analysis indicated 90% RH was chosen significantly less often than the 30% RH (*p* = 0.0009), 5% RH (*p* = 0.0016), or 60% RH airstreams (*p* = 0.0082) (Figure 3a). Moisture levels affected percent of time nymphs spent in the zones delimited near the ports (F_3,120_ = 6.18, *p* = 0.0006) (Figure 3b) but not total time insects spent moving (F_3,40_ = 0.93, *p* = 0.4353).

Nymphs spent significantly more time in zones near the 30% RH airstream (30.4% of trial time) than in saturated airstreams of 60% (13% of trial time) or 90% RH (2.7% of trial time) (Figure 3b). No significant differences were observed in time spent in zones near 5% RH and 30% RH, 5% RH and 60% RH, and 60% RH and 90% RH (Figure 3b) (*p* > 0.05). No statistically significant differences were detected in the percent of time insects spent moving (range: 21–44.3%) (F_3,40_ = 0.93, *p* = 0.435) or angular velocity (range 87.7–110 deg/s) (F_3,40_ = 0.3, *p* = 0.825). 

### 3.3. Responses to Light

**Figure 4:**
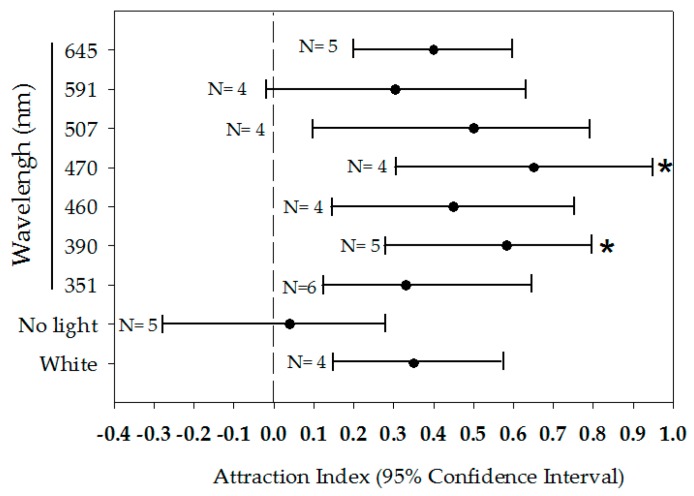
95% confidence intervals of attraction index means of *Triatoma rubida* males to different light wavelengths. Bootstrap analysis reveals that wavelengths of 470 and 390 nm are significantly more attractive than the control (no light) (*p* > 0.05).

*Triatoma rubida* adults responded to all wavelengths, including red and yellow. None of the attraction index means were less than 0.30; they were all greater than the index value of the control trials (mean A.I. = 0.04) (Figure 4). Bootstrap analysis reveals that 470 nm light had the highest attraction index value (mean A.I. = 0.65) and was significantly different from the control (no light) (mean A.I. = 0.04) (95% CI = 0.30–0.95 and −0.28–0.28, respectively). With a mean A.I. of 0.58 (95% CI = 0.29–0.79), 390 nm light was also significantly more attractive than the control (no light) (Figure 4). 

## 4. Discussion

*Triatoma rubida* inhabit environments in close association with their hosts, which include wild and domestic animals, as well as humans. It would not be surprising to find these insects travelling short distances in search of food. In these environments, host-derived cues such as heat, body odors, CO_2_, and moisture could play an important role during the orientation process [37]. Heat is a particularly important cue at short distances, guiding orientation behavior and inducing probing behavior in kissing bugs [38,39]. These short-range responses to heat are observed in the laboratory when *T. rubida* orient and probe heated membranes used for artificial feeding [40]. Several odors emitted by hosts, including CO_2_, have been implicated as an olfactory cue for virtually all blood-sucking insects [41], including kissing bugs [28,42,43]. A human host produces approximately 4500 ppm of CO_2_ through breath, but these levels decrease exponentially with distance [44]. Kissing bugs may sense changes in CO_2_ concentrations rather than constant concentrations since they likely encounter a gradient of CO_2_ near their hosts.

Locomotor responses of *T. rubida* to airstreams carrying different levels of CO_2_ were characterized with a four-port olfactometer. Four airstreams were directed toward a central chamber where orientation and locomotor activity of individual insects were recorded. The olfactometer system was supplied with air actively mixed on-site using industrial-grade air tanks, as opposed to adding CO_2_ to compressed and filtered atmospheric air, as in the study by Barrozo and Lazzari [44]. This allowed the creation of highly precise concentrations of CO_2_ in the olfactometer unaffected by ambient CO_2_. In initial experiments, nymphs of *T. rubida* that were exposed simultaneously to four airstreams artificially loaded with different levels of CO_2_ displayed upwind responses to airstreams carrying 1600 ppm and 3200 ppm of CO_2_. Locomotor activity of *T. rubida* in each odor zone was further characterized with the analysis of deviation of insects on a path to the ports that contained CO_2_-loaded airstreams. Overall, insects deviated less to airstreams carrying CO_2_ than airstreams carrying clean air (0 ppm of CO_2_). Furthermore, two of the tested CO_2_ concentrations, 1600 ppm and 3200 ppm, had the least deviation overall, confirming that these concentrations are the most influential on orientation responses from *T. rubida*. Carbon dioxide did not elicit an increase in activity or walking velocity in *T. rubida*, and these results are consistent with those reported by other researchers in experiments with other kissing bugs [42,44,45]. However, we cannot exclude the possibility that *T. rubida* might increase their locomotor activity when airstreams of CO_2_ are presented in a different fashion. Interestingly, some insects chose ports from which airstreams carried no stimuli, and this finding is not surprising as kissing bugs exhibit spontaneous orientation to odorless airstreams [25]. Even clean but consistently moving airstreams can act as a signal that alerts bugs to the presence of a potential host, at least initially. Positive anemotactic responses to these currents by insects could increase their chance to encounter potential cues signaling a host [25]. 

Concentrations of CO_2_ that elicited orientation responses from *T. rubida* were at least four times higher than ambient concentrations of CO_2_ (~400 ppm) [46]. Therefore, our data confirm that CO_2_ is an attractant for kissing bugs. Data reported here also indicate that *T. rubida* detects changes in CO_2_ relative to ambient CO_2_. Many insect species sense CO_2_ variations by olfactory sensilla on their mouthparts, legs, and antenna [47,48]. Specific CO_2_ receptors in blood-feeding flies respond to an increase in concentration rather than a discrete concentration of CO_2_ [49,50]. Kissing bugs have olfactory sensilla on the surface of their antennae that respond to several bioactive host-odor components [51]. However, these sensillae, as well as the olfactory sensory neurons (OSNs) involved in CO_2_ detection, have not been characterized yet. Ongoing work on antennal transcriptomes of *T. rubida* identified putative odorant receptors (OR) including odorant receptor co-receptor (ORCO), odorant-binding proteins (OBP), and gustatory receptors [52]. Whether or not these receptors, proteins, and their associated olfactory structures are involved in odor or CO_2_ detection needs to be further investigated. 

Comparison of these results with those from other studies should be done with caution due to differences in methodologies. For example, Barrozo and Lazzari [44] evaluated responses of *T. infestans* to CO_2_ in a locomotor compensator set in an open area. Since insects in this system were exposed to both CO_2_ testing levels and ambient CO_2_ (300–400 ppm), some adjustments are required for comparative purposes. That being said, results reported here suggest the behavioral sensitivity of *T. rubida* to CO_2_ is lower than that reported for *T. infestans* [44]. While a concentration of CO_2_ of 800 ppm was not preferred over 0 ppm by *T. rubida* here, 800 ppm was the threshold concentration that elicited responses from *T. infestans* [44]. In contrast, a higher sensitivity to CO_2_ occurs in the tsetse fly (*Glossina morsitans*) [53], mosquitoes [54], and stable flies [50], where sudden changes in CO_2_ concentration relative to ambient air elicit orientation responses.

Orientation responses elicited from *T. rubida* resulted solely from the addition of CO_2_ into a continuous airstream. However, sensitivity of insects to CO_2_ is not invariable and can be enhanced by the presence of other chemical cues. In mosquitoes, sensitivity thresholds to CO_2_ decrease when l-lactic acid was also presented [55], and similar synergist effects were observed in *T. infestans* [56] and in *Rhodnius prolixus* [57]. Further evaluation of the potential synergistic effect of CO_2_ with other stimuli would facilitate the development and deployment of monitoring or controlling devices (traps) for *T. rubida*. In addition, future work should also evaluate long-range responses of *T. rubida* to CO_2_ (and synergistic mixtures) to determine the range at which these odors can be detected. 

Water vapor has been implicated in the localization of vertebrate hosts by blood-sucking insects, but most studies have been restricted to the electrophysiological or morphological properties of sense organs [58,59]. The ability of blood-feeding insects to detect and respond to humidity gradients at long and short ranges is still poorly understood. Insects can exhibit hygropreference by reducing their speed (negative orthokinesis) and turning movements (negative klinokinesis) in the preferred humidity zone [60]. In our study, velocities and angular velocities of insects in each humidity zone were not significantly different. However, analysis of choices made by bugs and time these bugs spent in each zone indicated that hygropreference occurs in *T. rubida*, which preferred airstreams carrying 30% RH. Our findings coincide with previous observations in a related species, *T. infestans* [25]. These authors reported that the water alone orients insects to moisture-carrying airstreams. However, contrary to our findings, *T. infestans* did not display preferential orientation responses for airstreams of 30% and 70% RH (when compared with 60% or 70% RH) [25]. In our study, the response of *T. rubida* to relative humidities above 30% diminished as humidity increased. *T. rubida* preference to 30% RH suggests this species senses changes to humidity in their environment. This preference might be relevant not only during host seeking but also for other important biological events such as harboring, molting, and oviposition. These events optimally take place at low relative humidity in other kissing bugs [61,62]. Although there is no information on morphology and physiology of hygroreceptors in kissing bugs, this type of receptor has been identified on the antennae of cockroaches and stick insects, which harbor humid and dry cells that respond to increments or decrements of relative humidity [59]. From a practical point of view, orientation responses to water vapor could be exploited for the development of a trapping system for *T. rubida*. Concomitant use of water vapor and heat, or water vapor with CO_2_, would likely decrease the response thresholds of the insects and present more attractive stimuli at greater distances than water vapor alone. Hygropreference results of this study have crucial implications not only for trap development but also for behavioral bioassays that use airstreams to deliver stimuli. *Triatoma rubida* never chose the airstream with a high humidity content (90% RH). Nymphs spent very little time near the port. In fact, during trials nymphs would quickly turn around after venturing into the 90% RH zone, suggesting that high humidity airstreams repel these bugs. When performing olfactometer studies it is commonplace to humidify airstreams, as conducted in Milne et al. [24], in order to assist in carrying odor molecules or heat and facilitate its interaction with sensory receptors. Our data presents ample reason to reexamine this practice and consider changing it. Humidified airstreams, which are about 90% RH, may interfere when attempting to demonstrate preference for an odorant. This potential problem should be investigated as novel test organisms are introduced to olfactometer bioassays.

Analysis of attraction to light of different wavelengths showed that adult male *T. rubida* were attracted to most wavelengths of light, including red (645 nm), but that there was a preference for short wavelengths. Of all the wavelengths, violet light (470 nm) achieved the strongest attractive response. Surprisingly, it was found that light of wavelength 351 nm, which is much further in the UV range, was not attractive. This represents the first time that such a short wavelength or any wavelength in the UV range has been tested with kissing bugs (the shortest wavelength tested in Pacheco-Tucuch et al. [12] was 430 nm). In the psocid *Liposcelis bostrychophila* this wavelength (351 nm) elicited strong attractive responses [33]. Our results coincide with previous light sensitivity studies with the related species *T. dimidiata* that reported high attraction to blue (430 nm) with decreased attraction to increasing wavelengths [12]. In contrast, Reisenman et al. [63], in a behavioral context of assembling, evaluated spectral light sensitivity of *T. infestans* and found that bugs aggregated in both blue (short wavelength) and red (long wavelength) areas. Interestingly, *T. infestans* consistently displayed photonegative reaction to the long wavelength green. The disparity in light sensitivities may represent key differences between southwestern kissing bugs and those encountered in Latin America that could be shaped by the natural history of species in each environment. The differential responses to the spectrum of light among kissing bugs also imply the presence of chromatic mechanisms in insects’ photoreceptors that help to discriminate wavelengths. Although the existence of such photoreceptors in kissing bugs has been not demonstrated, their presence might explain avoidance reactions to a green portion of the spectrum by *T. infestans* observed during Reisenman’s study [63] and the diminished attraction of *T. rubida* to ultraviolet (351 nm) and yellow (591 nm) light observed during our study. The attractive response to white light was present but not formidable. As with Pacheco-Tucuch et al. [12], white light was not a stronger attractant than some wavelengths alone. One possible reason for the low response to white light is that it contains all the wavelengths of visible light, and therefore will not be as attractive as the most attractive wavelength (470 nm).

Perhaps more interesting than the attractiveness of violet light was the attractiveness of red light (645 nm), which was completely unexpected. Far more bugs stayed in the arena during these trials and no bugs whatsoever chose the control pitfall (no light). This could result from the relative weakness of red light, being attractive enough to keep bugs in the arena but not necessarily enough to choose the treatment. It should be noted that our study is quite limited by its scale. The scale of the experiment performed (diameter = 14.5 cm) is wholly inadequate to simulate natural conditions of dispersal flights. An experiment that allows or requires kissing bugs to fly in a larger arena in order to make a choice would provide supplemental information that could be used to develop an effective light-based trap for *T. rubida.* Future studies should also include synergistic effect of multiple cues and evaluations of long-range responses. An understanding of these responses would contribute to the development of a trapping system for *T. rubida*. 

## 5. Conclusions

This study demonstrates that *T. rubida* detect a great variety of stimuli, such as CO_2_, moisture and light. The preference to 1600 and 3200 ppm CO_2_, low levels of humidity and violet light suggests that there exists a functional sensory system that allows *T. rubida* to perceive different levels of stimuli. The study of orientation responses of *T. rubida* to host-associated stimuli increases not only our knowledge about the sensory capabilities of these epidemiologically important insects, but also contributes to the development of methods that can be used for monitoring *T. rubida* in areas where this species is present.

## References

[1] Klotz J.H., Dorn P.L., Logan J.L., Stevens L., Pinnas J.L., Schmidt J.O., Klotz S.A. (2010). “Kissing bugs”: Potential disease vectors and cause of anaphylaxis. Clin. Infect. Dis..

[2] Roberts L.S., Janovy J., Nadler S. (2013). Foundations in Parasitology.

[3] Lee B.Y., Bacon K.M., Bottazzi M.E., Hotez P.J. (2013). Global economic burden of Chagas disease: A computational simulation model. Lancet Infect. Dis..

[4] Garza M., Feria Arroyo T.P., Casillas E.A., Sanchez-Cordero V., Rivaldi C.-L., Sarkar S. (2014). Projected future distributions of vectors of *Trypanosoma cruzi* in North America under climate change scenarios. PLoS Negl. Trop. Dis..

[5] Reisenman C.E., Lawrence G., Guerenstein P.G., Gregory T., Dotson E., Hildebrand J.G. (2010). Infection of kissing bugs with *Trypanosoma cruzi*, Tucson, Arizona, USA. Emerg. Infect. Dis..

[6] Martínez-Ibarra J.A., Paredes-González E., Licón-Trillo A., Montañez-Valdez O.D., Rocha-Chávez G., Nogueda-Torres B. (2012). The biology of three Mexican-American species of Triatominae (Hemiptera: Reduviidae): *Triatoma recurva*, *Triatoma protracta* and *Triatoma rubida*. Mem. Inst. Oswaldo Cruz.

[7] Reisenman C.E., Savary W., Cowles J., Gregory T.L., Hildebrand J.G. (2012). The distribution and abundance of Triatomine insects, potential vectors of Chagas disease, in a metropolitan area in Southern Arizona, United States. J. Med. Entomol..

[8] Klotz S.A., Dorn P.L., Mosbacher M., Schmidt J.O. (2014). Kissing bugs in the United States: Risk for vector-borne disease in humans. Environ. Health Insights.

[9] Ryckman R.E. (1986). The vertebrate hosts of the Triatominae of North and Central America and the West Indies (Hemiptera: Reduviidae: Triatominae). Bull. Soc. Vector Ecol..

[10] Wood S.F. (1941). Notes on the distribution and habits of Reduviid vectors of Chagas disease in the Southwest United States. Pan Pac. Entomol..

[11] Wood S.F. (1950). Dispersal flight of *Triatoma* in southern Arizona. J. Parasitol..

[12] Pacheco-Tucuch F.S., Ramirez-Sierra M.J., Gourbière S., Dumonteil E. (2012). Public street lights increase house infestation by the Chagas disease vector *Triatoma dimidiate*. PLoS ONE.

[13] Zeledon R.B.C., Pinto D.J., Leiby D., Dorn P., Coura J.R. (2012). An Appraisal of the Status of Chagas Disease in the United States.

[14] Walter J., Fletcher E., Moussaoui R., Gandhi K., Weirauch C. (2012). Do bites of kissing bugs cause unexplained allergies? Results from a survey in triatomine-exposed and unexposed areas in Southern California. PLoS ONE.

[15] Klotz S.A., Mazda Shirazi F., Boesen K., Beatty N.L., Dorn P.L., Smith S., Schmidt J.O. (2016). Kissing bug (*Triatoma* spp.) intrusion into homes: Troublesome bites and domiciliation. Environ. Health Insights.

[16] Sfara V., Zerba E.N., Alzogaray R.A. (2006). Toxicity of pyrethroids and repellency of diethyltoluamide in tow deltamethrin-resistant colonies of *Triatoma infestans* Klug, 1834 (Hemiptera: Reduviidae). Mem. Inst. Oswaldo Cruz.

[17] Rozendaal J.A. (1997). Triatomine bugs. Vector Control. Methods for Use by Individuals and Communities.

[18] Buhaya M.H., Galvan S., Maldonado R.A. (2015). Incidence of *Trypanosoma cruzi* infection in triatomines collected at Indio Mountains Research Station. Acta Trop..

[19] Stevens L., Dorn P.L., Hobson J., de la Rua N.M., Lucero D.E., Klotz J.H., Schmidt J.O., Klotz S.A. (2012). Vector blood meals and Chagas disease transmission potential, United States. Emerg. Infect. Dis..

[20] Pierce J. (2017). Personal communication.

[21] Reisenman C.E., Gregory T., Guerenstein P.G., Hildebrand J.G. (2011). Feeding and defecation behavior of *Triatoma rubida* (Uhler, 1894) (Hemiptera: Reduviidae) under laboratory conditions, and its potential role as a vector of Chagas disease in Arizona, USA. Am. J. Trop. Med. Hyg..

[22] Lazzari C.R., Lorenzo M.G. (2009). Exploiting triatomine behaviour: Alternative perspectives for their control. Mem. Inst. Oswaldo Cruz.

[23] Wang C., Gibb T., Bennett G.W., McKnight S. (2009). Bed bug (Heteroptera: Cimicidae) attraction to pitfall traps baited with carbon dioxide, heat, and chemical lure. J. Econ. Entomol..

[24] Milne M.A., Ross E.J., Sonenshine D.E., Kirsch P. (2009). Attraction of *Triatoma dimidiata* and *Rhodnius prolixus* (Hemiptera: Reduviidae) to combinations of host cues tested at two distances. J. Med. Entomol..

[25] Barrozo R.B., Manrique G., Lazzari C.R. (2003). The role of water vapour in the orientation behaviour of the blood-sucking bug *Triatoma infestans* (Hemiptera, Reduviidae). J. Insect Physiol..

[26] Lazzari C.R. (2009). Orientation towards hosts in haematophagous insects: An integrative perspective. Adv. Insect Physiol..

[27] Gillies M.T. (1980). The role of carbon dioxide in host-finding by mosquitoes (Diptera: Culicidae): A review. Bull. Entomol. Res..

[28] Guerenstein P.G., Lorenzo M.G., Núñez J.A., Lazzari C.R. (1995). Baker's yeast, an attractant for baiting traps for Chagas’ disease vectors. Experientia.

[29] Shimoda M., Honda K.-I. (2013). Insect reactions to light and its applications to pest management. App. Entomol. Zool..

[30] Anderson J.F., Ferrandino F.J., McKnight S., Nolen J., Miller J. (2009). A carbon dioxide, heat and chemical lure trap for the bedbug, *Cimex lectularius*. Med. Vet. Entomol..

[31] Montes C., Cuadrillero C., Vilella D. (2002). Maintenance of a laboratory colony of *Cimex lectularius* (Hemiptera: Cimicidae) using an artificial feeding technique. J. Med. Entomol..

[32] Noldus L.P.J.J., Spink A.J., Tegelenbosch R.A.J. (2002). Computerised video tracking, movement analysis and behaviour recognition in insects. Comput. Electron. Agric..

[33] Diaz-Montano J., Campbell J.F., Phillips T.W., Cohnstaedt L.W., Throne J.E. (2015). Evaluation of light attraction for the stored-product psocid, *Liposcelis bostrychophila*. J. Pest Sci..

[34] Sharpe D. (2015). Your Chi-Square test is statistically significant: Now what?. Pract. Assess. Res. Eval..

[35] Efron B., Tibshirani R.J. (1998). An Introduction to the Bootstrap.

[36] SAS Institute Inc. (2013). Base SAS® 9.4 Procedures Guide: Statistical Procedures.

[37] Barrozo R.B., Reisenman C.E., Guerenstein P., Lazzari C.R., Lorenzo M.G. (2017). An inside look at the sensory biology of triatomines. J. Insect Physiol..

[38] Lazzari C.R., Núñez J.A. (1989). Blood temperature and feeding behavior in *Triatoma infestans* (Heteroptera: Reduviidae). Entomol. Gen..

[39] Ferreira R.A., Lazzari C.R., Lorenzo M.G., Pereira M.H. (2007). Do haematophagous bugs assess skin surface temperature to detect blood vessels?. PLoS ONE.

[40] Indacochea A. (2017). Personal communication.

[41] Lehane M.J. (2005). The Biology of Blood-Sucking in Insects.

[42] Taneja J., Guerin P.M. (1995). Oriented responses of the triatomine bugs *Rhodnius prolixus* and *Triatoma infestans* to vertebrate odours on a servosphere. J. Comp. Physiol. A.

[43] Taneja J., Guerin P.M. (1997). Ammonia attracts the haematophagous bug *Triatoma infestans*: Behavioural and neurophysiological data on nymphs. J. Comp. Physiol. A.

[44] Barrozo R.B., Lazzari C.R. (2004). The response of the blood-sucking bug *Triatoma infestans* to carbon dioxide and other host odours. Chem. Senses.

[45] Núñez J.A. (1982). Food source orientation and activity in *Rhodnius prolixus*. Bull. Entomol. Res..

[46] Guerenstein P.G., Hildebrand J.G. (2007). Roles and effects of environmental carbon dioxide in insect life. Annu. Rev. Entomol..

[47] Stange G., Stowe S. (1999). Carbon-dioxide sensing structures in terrestrial arthropods. Microsc. Res. Tech..

[48] Omondi B.A., Majeed S., Ignell R. (2015). Functional development of carbon dioxide detection in the maxillary palp of *Anopheles gambiae*. J. Exp. Biol..

[49] Sage R.F. (2002). How terrestrial organisms sense, signal, and respond to carbon dioxide. Integr. Comp. Biol..

[50] Warnes M.L., Finlayson L.H. (1986). Electroantennogram responses of the stable fly, *Stomoxys calcitrans*, to carbon dioxide and other odours. Physiol. Entomol..

[51] Guerenstein P.G., Guerin P.M. (2001). Olfactory and behavioural responses of the blood-sucking bug *Triatoma infestans* to odours of vertebrate hosts. J. Exp. Biol..

[52] Romero A. (2017).

[53] Bogner F. (1992). Response properties of sensitive receptors in tsetse flies (Diptera: *Glossina palpalis*). Physiol. Entomol..

[54] Kellogg F.E. (1970). Water vapor and carbon dioxide receptors in *Aedes aegypti*. J. Insect Physiol..

[55] Acree F.J., Turner R.B., Gouck H.K., Beroza M., Smith N. (1968). l-lactic acid: A mosquito attractant isolated from humans. Science.

[56] Barrozo R.B., Lazzari C.R. (2004). Orientation behaviour of the blood-sucking bug *Triatoma infestans* to short-chain fatty acids: Synergistic effect of l-Lactic acid and carbon dioxide. Chem. Senses.

[57] Otalora-Luna F., Perret J.L., Guerin P.M. (2004). Appetence behaviours of the triatomine bug *Rhodnius prolixus* on a servosphere in response to the host metabolites carbon dioxide and ammonia. J. Comp. Physiol. A.

[58] Yokohari F., Tateda H. (1976). Moist and dry hygroreceptors for relative humidity of the cockroach, *Periplaneta americana* L.. J. Comp. Physiol..

[59] Tichy H., Kallina W. (2010). Insect hygroreceptor responses to continuous changes in humidity and air pressure. J. Neurophysiol..

[60] Evans W.G. (1997). Humidity-invoked upwind orientation of shore insects (*Bambidion obtusidens*, Coleoptera: Carabidae). J. Insect Behav..

[61] Roca M.J., Lazzari C.R. (1994). Effects of relative humidity on the hematophagous bug *Triatoma infestans*: Hygropreference and eclosion success. J. Insect Physiol..

[62] Guarneri A.A., Lazzari C., Diotaiuti L., Lorenzo M.G. (2002). The effect of relative humidity on the behaviour and development of *Triatoma brasiliensis*. Physiol. Entomol..

[63] Reisenman C.E., Lorenzo Figueiras A.N., Giurfa M., Lazzari C.R. (2000). Interaction of visual and olfactory cues in the aggregation behaviour of the haematophagous bug *Triatoma infestans*. J. Comp. Physiol. A.

